# Primary aortoenteric fistula: A case report and brief review of the literature

**DOI:** 10.1016/j.radcr.2021.08.044

**Published:** 2021-09-14

**Authors:** Dhairya A. Lakhani, Shantosh A. Sharma, Haneen Kutayni, Aneri B. Balar, Gary Marano, Daniel Martin

**Affiliations:** Department of Radiology, West Virginia University, Ruby Memorial Hospital, 1 Medical Center Drive, Morgantown, WV 26506, USA

**Keywords:** Aortoenteric fistula, Axillobifemoral bypass, Acute aortic syndrome

## Abstract

Aortoenteric fistula is a life-threatening emergency and is associated with high morbidity and mortality. Prompt surgical intervention before the aneurysm ruptures lowers the mortality rate to about 50%. Potential imaging mimics for aortoenteric fistula include retroperitoneal fibrosis, mycotic aortic aneurysm, and infectious aortitis. Secondary aortoenteric fistula has relative higher incidence compared to primary and is more common with open aortic repair versus endovascular stent graft repair. Ectopic gas in the aneurysm sac and extravasation of enteric contrast into the aneurysm sac is diagnostic for aortoenteric fistula. However, enteric contrast is not recommended for routine evaluation of aortoenteric because the aforementioned finding is extremely rare. More common imaging findings include bowel loop appearing adherent to aneurysm sac with associated inflammatory stranding and foci or ectopic gas within the aneurysm sac or interposed between the bowel and aneurysm sac. Here we present a case of 52-year-old male who presents with incidental primary aortoenteric fistula.

## Background

Aortoenteric fistula is defined as an abnormal communication between the aorta or aortoiliac tree with the gastrointestinal tract and presents as catastrophic gastrointestinal bleeding with annual incidence of primary aortoenteric fistula reported at 0.007 per million (with 250 cases reported in literature) [1,2]. There is relatively higher prevalence of secondary aortoenteric fistula following open aortic repair compared to endovascular stent graft repair of the abdominal aorta [1,3].

Aortoenteric fistula present as minor "herald" gastrointestinal bleeding followed by later catastrophic life-threatening gastrointestinal hemorrhage [1,4]. May present with recurrent septicemia from enteric pathogen and abdominal pain similar to aortitis. Some patients may present with abdominal pain and pulsatile sensation over the abdomen [1,^2^,^5^,6].

Here, we present a case of aortoenteric fistula who underwent open resection of abdominal aortic aneurysm with a right axillary to bifemoral bypass graft.

## Case description

**Fig. 1:**
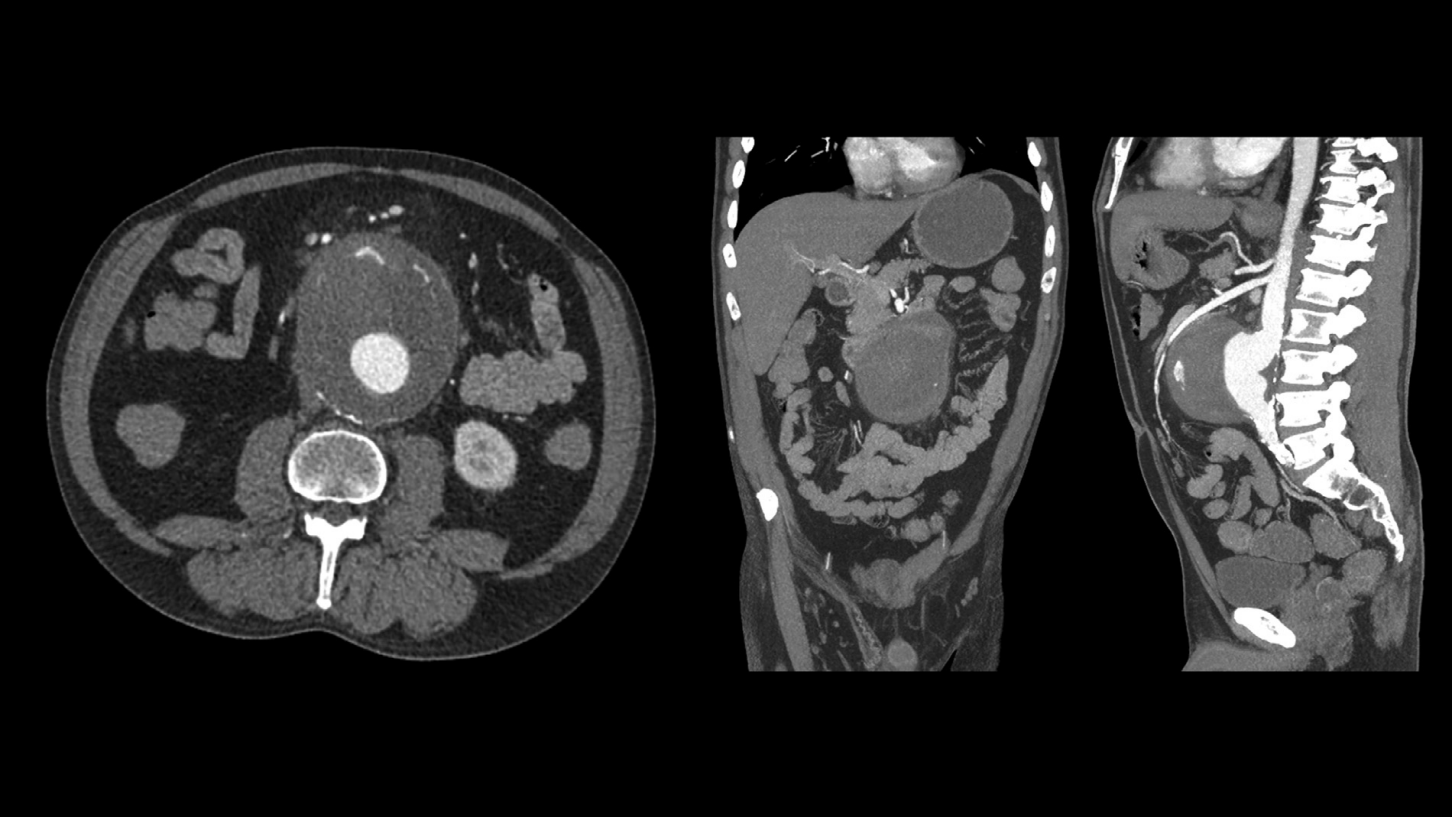
CT abdomen and pelvis with contrast was performed which showed infrarenal abdominal aortic aneurysm measuring 8.9 × 10.3 cm.

A 52-year-old Caucasian male presented to the outside facility emergency department following a syncopal episode and dysthymia. On arrival his vital signs were within normal limits. No leukocytosis. Chest radiograph and unenhanced CT brain were normal. CT abdomen and pelvis with contrast was performed which showed infrarenal abdominal aortic aneurysm measuring 8.9 × 10.3 cm (Fig. 1).

**Fig. 2:**
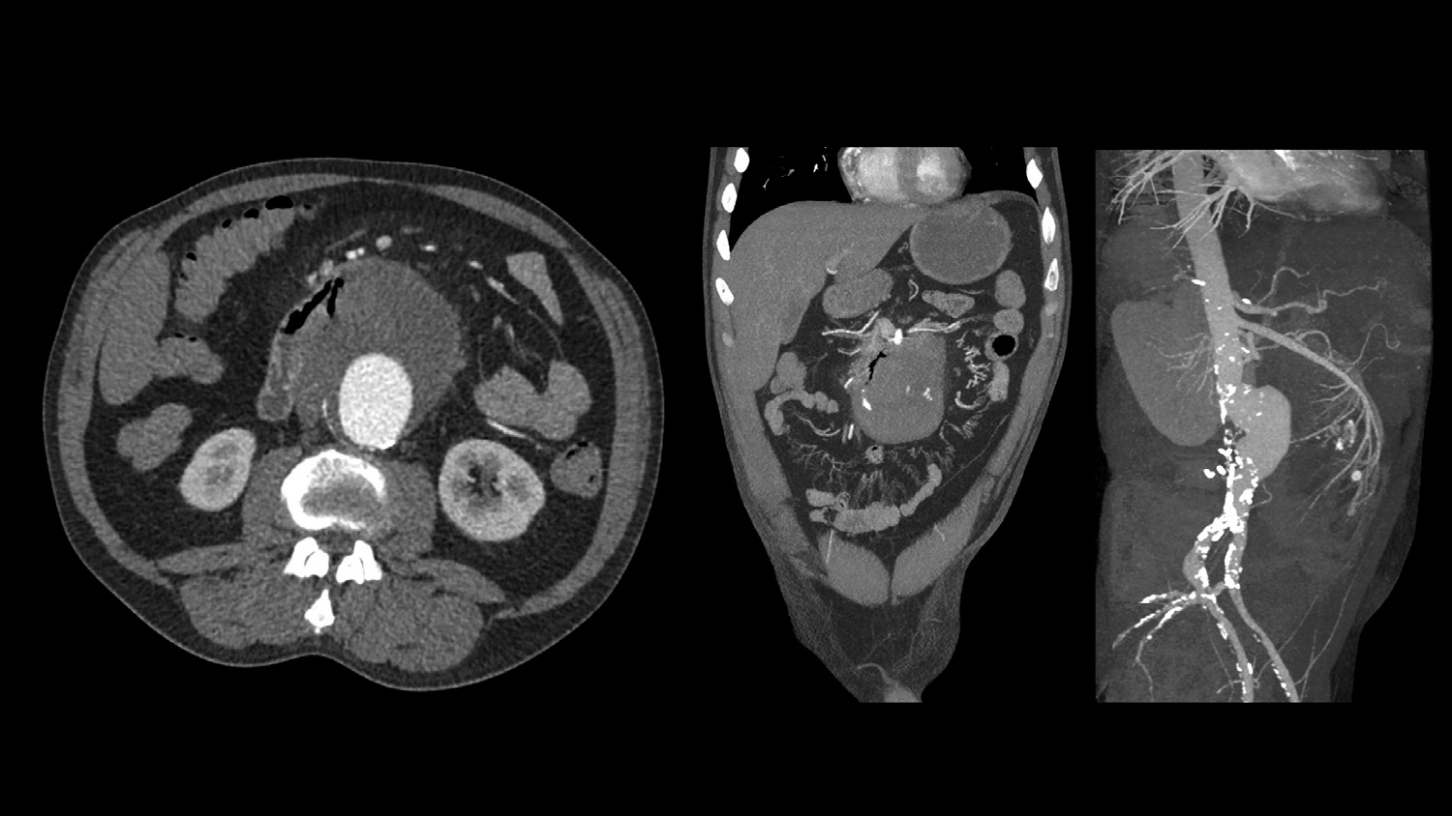
Additionally, the thrombosed infrarenal abdominal aortic aneurysm demonstrated intramural air and was in close approximation to the proximal jejunum, findings compatible with primary aorto-enteric fistula. Of note: This was noted on retrospect at outside following aortobiiliac stent graft placement at outside facility (Fig. 3).

Additionally, on retrospect, the loop of jejunum appeared adherent to aneurysm sac, with ectopic air within the partially thrombosed aneurysm sac tracking to and in continuity with intraluminal bowel gas. Findings compatible with primary aortoenteric fistula (Fig. 2).

**Fig. 3:**
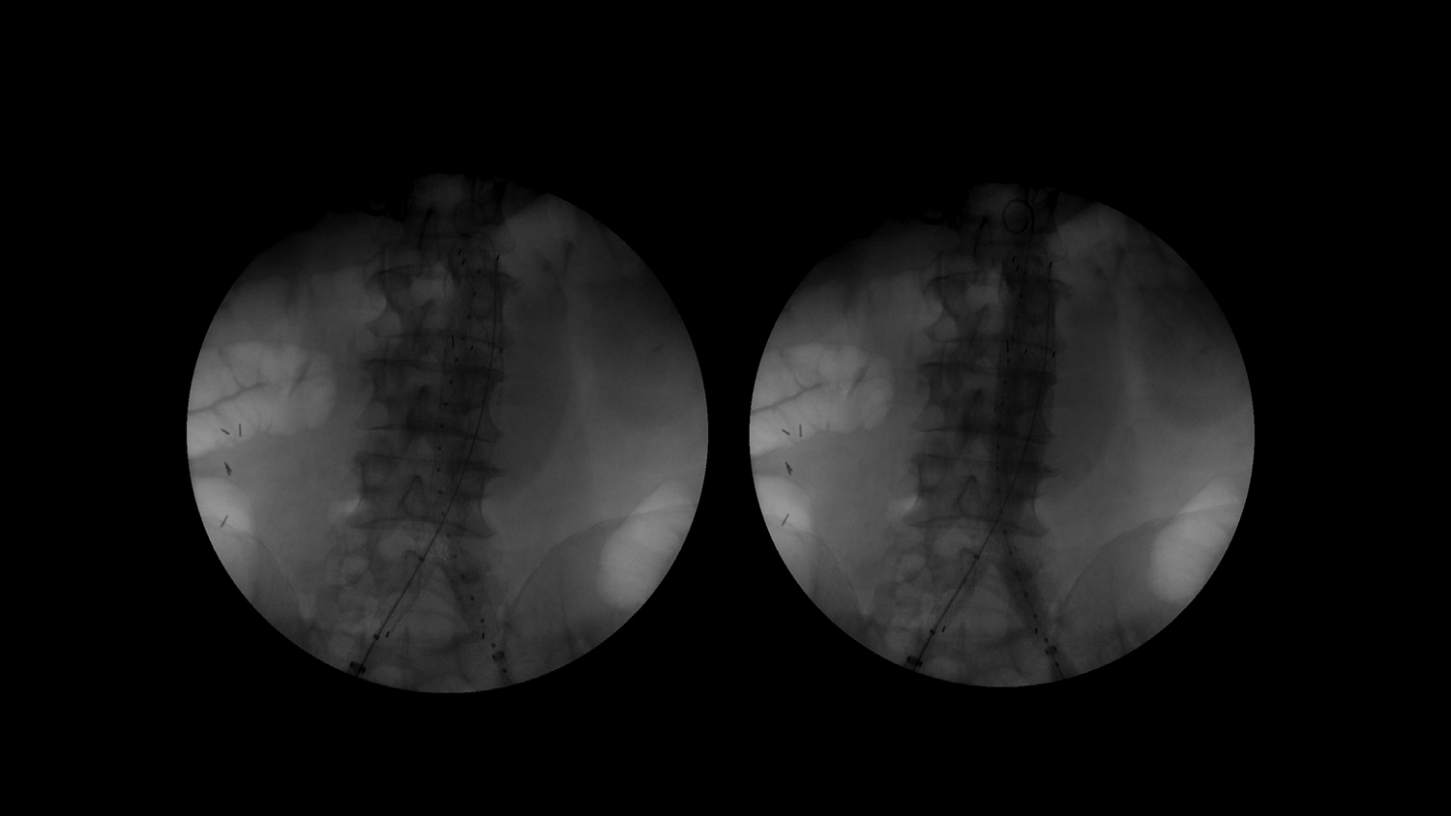
Patient underwent endovascular aortobiiliac stent graft placement on the same day. Fluoroscopy imaging demonstrates no evidence of endoleak and appropriate position of the aortobiiliac stent.

Patient underwent endovascular aortobiiliac stent graft placement on the same day at outside facility (Fig. 3). Fluoroscopy imaging demonstrate no evidence of endoleak and appropriate position of the aortobiiliac stent.

**Fig. 4:**
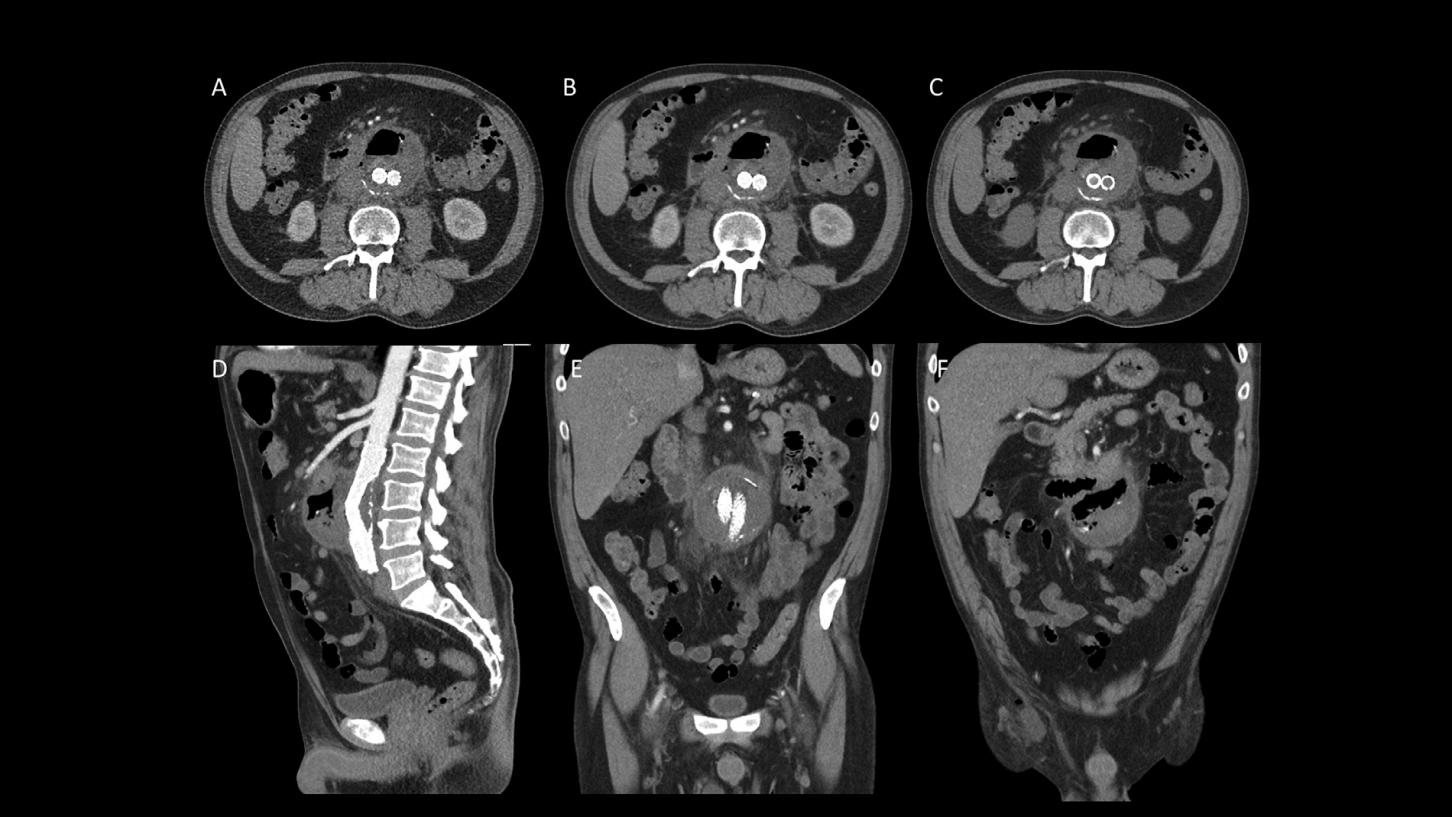
Five days following the procedure patient reported bright red blood per rectum with stable vitals and hematocrit. CT angiogram of the abdomen and pelvis was performed. Arterial phase (A, D and E), delayed phase (B) and unenhanced study (C and F). It showed normal positioning of aortobiiliac stent graft without evidence of endoleak. Stable size of the excluded aneurysmal sac. There was interval worsening of ectopic gas within the excluded aneurysmal sac. Findings compatible with known aortoenteric fistula.

Five days following the procedure patient reported bright red blood per rectum with stable vitals and hematocrit. CT angiogram of the abdomen and pelvis was performed which showed normal positioning of aortobiiliac stent graft and no evidence of endoleak. The size of the excluded aneurysmal sac was stable from prior exam. There was interval worsening of ectopic air within the excluded aneurysmal sac. Findings were compatible with known aortoenteric fistula (Fig. 4).

**Fig. 5:**
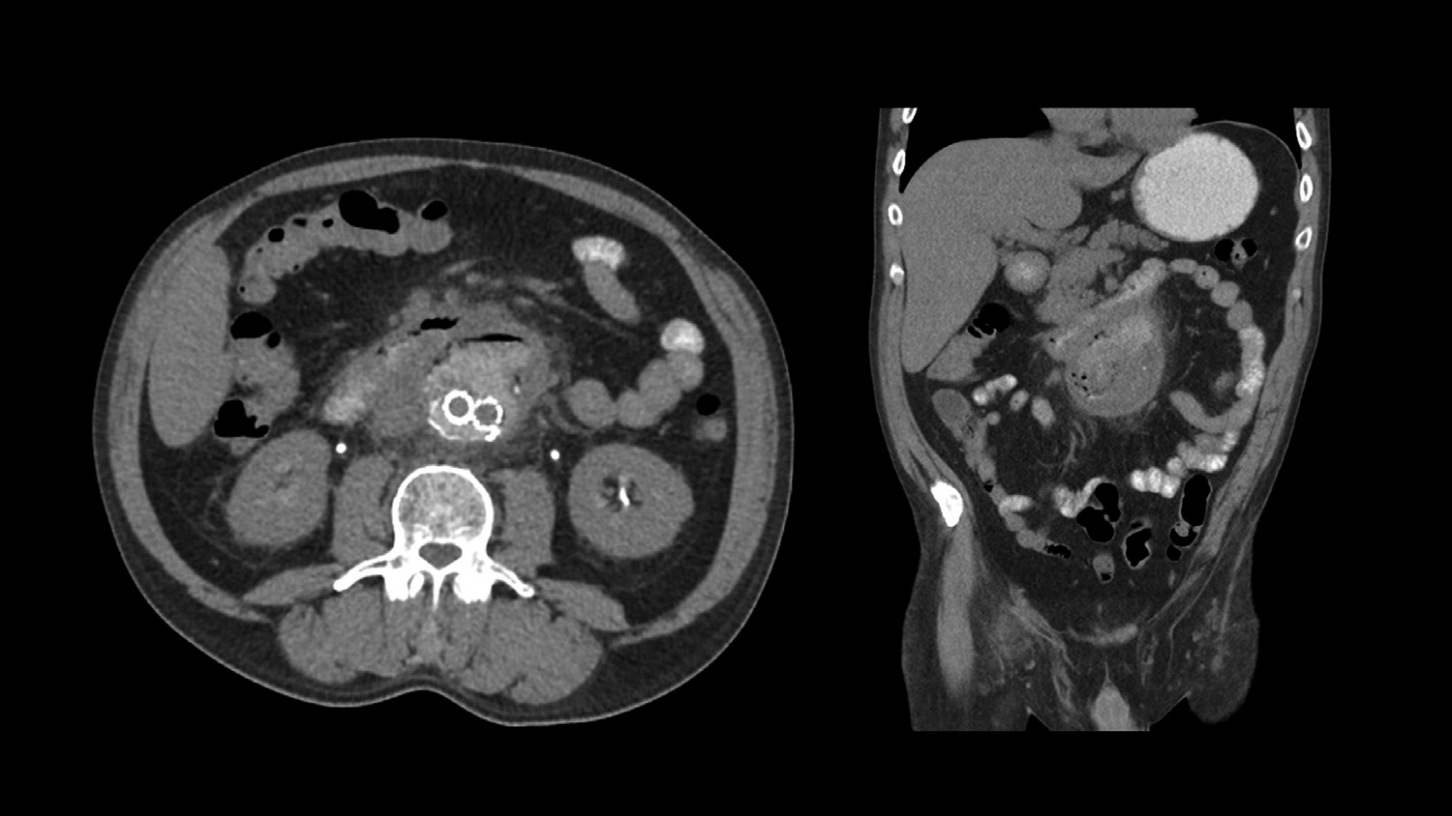
Prior to transfer, CT abdomen and pelvis with enteric contrast was performed which showed enteric contrast into the excluded aneurysmal sac, again confirming known aortoenteric fistula.

Additionally, prior to transfer at our facility, CT abdomen and pelvis without intravenous contrast was performed which showed enteric contrast into the excluded aneurysmal sac, confirming known aortoenteric fistula (Fig. 5).

**Fig. 6:**
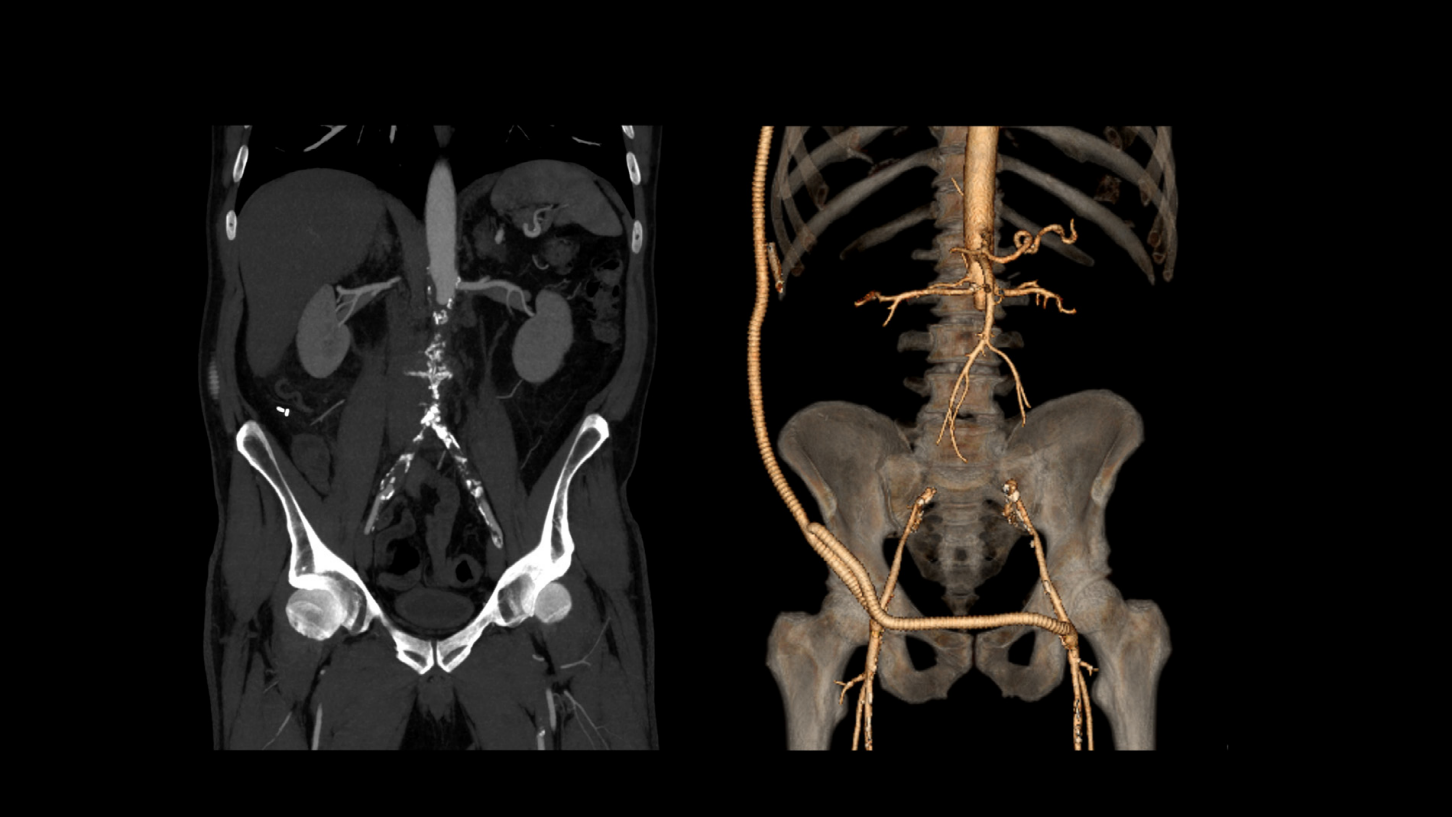
Patient underwent resection of abdominal aortic aneurysm with a right axillary to bifemoral bypass utilizing bifurcated ringed Polytetrafluoroethylene.

Patient was transferred to our facility for further management. Patient underwent resection of abdominal aortic aneurysm with a right axillary to bifemoral bypass utilizing bifurcated ringed Polytetrafluoroethylene, Figure 6.

## Discussion

Aortoenteric fistula was first described in 19th century by a British surgeon Sir Astley Cooper [7]. Aortoenteric fistula are classified as primary or secondary depending on the etiology. Primary aortoenteric fistula typically develops when abdominal or aortoiliac tree aneurysm closely abuts the bowel loops [2,^6^,8], and most commonly involving the distal duodenum and proximal jejunum due to long-standing pressure. The aneurysm then erodes into the bowel loop developing a fistulous connection. A secondary aortoenteric fistula can be seen with open aortic repair and less commonly with endovascular aortic stent graft repair [3]. These occur secondary to peri-graft infection and leak which occurs between 2 weeks and 10 years after the surgery [3,^6^,9].

CT findings of primary aortoenteric fistula includes presence of ectopic gas within the excluded aneurysmal sac and/or presence of enteric contrast in the aneurysm sac. Lack of fat plane between the vessel and bowel lumen may be early sign for developing aortoenteric fistula. Ancillary findings for secondary aortoenteric fistula includes peri-graft soft tissue inflammation, and adjacent bowel wall thickening [8].

Potential imaging mimics of aortoenteric fistula includes retroperitoneal fibrosis, mycotic aortic aneurysm, and infectious aortitis [8]. This is a surgical emergency and needs prompt surgical treatment. Operating mortality is reported as approximately 50% and mortality approaches 100% without any intervention [10].

## Patient consent

No patient identifiers are disclosed in current report.
